# Anti-placental growth factor antibody ameliorates hyperoxia-mediated impairment of lung development in neonatal rats

**DOI:** 10.1590/1414-431X20198917

**Published:** 2020-01-24

**Authors:** Zhiqun Zhang, Ying Zhong, Xiaoxia Li, Xianmei Huang, Lizhong Du

**Affiliations:** 1 Department of Neonatology, Affiliated Hangzhou First People's Hospital, Zhejiang University School of Medicine, Hangzhou, Zhejiang, China; 2 Department of Neonatology, the Children's Hospital, Zhejiang University School of Medicine, Hangzhou, Zhejiang, China

**Keywords:** Bronchopulmonary dysplasia, Anti-PGF antibody, Newborn rats, Hyperoxia, Lung tissue injury

## Abstract

This study investigates the effect of the overexpression of the placental growth factor (PGF) and hyperoxia on lung development and determines whether anti-PGF antibody ameliorates hyperoxia-mediated impairment of lung development in newborn rats. After exposure to normoxic conditions for seven days, newborn rats subjected to normoxia were intraperitoneally or intratracheally injected with physiological saline, adenovirus-negative control (Ad-NC), or adenovirus-PGF (Ad-PGF) to create the Normoxia, Normoxia+Ad-NC, and Normoxia+Ad-PGF groups, respectively. Newborn rats subjected to hyperoxia were intraperitoneally injected with physiological saline or anti-PGF antibodies to create the Hyperoxia and Hyperoxia+anti-PGF groups, respectively. Our results revealed significant augmentation in the levels of PGF and its receptor Flt-1 in the lung tissues of newborn rats belonging to the Normoxia+Ad-PGF or Hyperoxia groups. PGF overexpression in these groups caused lung injury in newborn rats, while anti-PGF antibody treatment significantly cured the hyperoxia-induced lung injury. Moreover, PGF overexpression significantly increased TNF-α and Il-6 levels in the bronchoalveolar lavage (BAL) fluid of the Normoxia+Ad-PGF and Hyperoxia groups. However, their levels were significantly reduced in the BAL fluid of the Hyperoxia+anti-PGF group. Immunohistochemical analysis revealed that PGF overexpression and hyperoxia treatment significantly increased the expression of the angiogenesis marker, CD34. However, its expression was significantly decreased upon administration of anti-PGF antibodies (compared to the control group under hyperoxia). In conclusion, PGF overexpression impairs lung development in newborn rats while its inhibition using an anti-PGF antibody ameliorates the same. These results provided new insights for the clinical management of bronchopulmonary dysplasia in premature infants.

## Introduction

Bronchopulmonary dysplasia (BPD) is a major cause of chronic lung disease and is a serious sequela in preterm infants (1). It causes poor developmental and clinical outcomes in very-low-birth-weight infants (weighing less than 1500 g by the age of 1 year) (2), resulting in long-term respiratory impairment and abnormal neurodevelopment (3). These consequences extend beyond childhood and result in increased health care costs. Effective therapies for BPD have not yet been developed (3).

BPD is characterized by aberrant pulmonary vascular growth and remodeling, arrested alveolar growth, and alveolar simplification (4–6). Abman proposed a vascular hypothesis, according to which disruption of angiogenesis during lung development can impair the normal growth of lungs, resulting in decreased alveolarization and pulmonary arterial density (7). Therefore, regulators of pulmonary angiogenesis may serve as therapeutic targets for BPD. Impaired vascular endothelial growth factor (VEGF) signaling has been associated with the pathogenesis of BPD in clinical settings. Placental growth factor (PGF) is an angiogenic factor belonging to the VEGF family (8,9). During normal pregnancy, the plasma PGF levels gradually increase from early pregnancy until weeks 29–30 and decrease thereafter (10). In addition, PGF levels are higher in the plasma of preterm neonates with BPD (11,12), suggesting an important role of PGF in vascular development in the lungs of preterm neonates with BPD. Therefore, inhibiting the function of PGF may help in the management of the abnormal pulmonary vascular development in BPD cases, consequently improving the symptoms of BPD.

Long-term exposure to hyperoxia is one of the critical factors in the development of BPD (13). In neonatal rats, prolonged exposure to hyperoxia reduces alveolar septation and increases terminal air space, which are characteristic features of BPD (14–16). Neonatal rats exposed to hyperoxia are used as animal models of BPD (17). It is reported that hyperoxia could enhance PGF release in the lungs of neonatal rats (18). Moreover, knockdown of PGF by lentiviral delivery mitigates hyperoxia-induced acute lung injury by suppressing the NFκB signaling pathway in neonatal rats (19). These results indicate that PGF plays a key role in regulating hyperoxia-mediated lung injury in neonatal rats. Therefore, investigating the mechanisms that inhibit the effects of PGF would prove useful for BPD treatment. In the present study, we aimed to determine whether an anti-PGF antibody can ameliorate hyperoxia-mediated impairment of lung development in newborn rats. In addition, we also investigated the effect of PGF overexpression on lung development in neonatal rats to further confirm the role of PGF in BPD.

## Material and Methods

### Animals

Pathogen-free-grade healthy Sprague-Dawley rats that were pregnant for 15 d were purchased from Shanghai SLRC Laboratory Animal Co., Ltd. (China). All animals were housed at room temperature (22±3°C) with a relative humidity of 60±5% and a 12-h light/dark cycle. Animals were raised in individual cages. All animal experiments were approved by the Hangzhou Hibio Animal Care and Use Committee (China).

### Adenoviral packaging

The full coding sequence (CDS) of PGF was cloned into a pDC316-mCMV-ZsGreen plasmid by DNA synthesis. The recombinant and pHelper packaging plasmids were co-transfected into HEK293A cells. The first- and second-generation adenoviruses were isolated and purified by cesium chloride density gradient ultracentrifugation at 20,000 *g* for 2 h at 4°C. The viral titers were determined by measuring the cytopathic effect on HEK293A cells in a 96-well plate using fluorescence microscopy. The negative control (NC)-empty adenoviruses were named Ad-NC, while the adenoviruses expressing PGF were named Ad-PGF.

### Anti-PGF antibody production

The full-length optimized CDS of PGF was cloned into the prokaryotic expression vector pGEX-6p-1 by DNA synthesis. The recombinant plasmid was transformed into the prokaryotic expression host *Escherichia coli* BL21(DE3). The expression of PGF protein was confirmed by SDS-PAGE (GenScript, China), and the protein was purified using a high-affinity Ni resin (GenScript) followed by renaturation using urea. The purified PGF protein was used to immunize male New Zealand white rabbits using the following protocol. Purified PGF protein was emulsified with Freund's complete adjuvant and injected multiple times into thigh muscles of the rabbits. After 28 days, purified PGF protein was emulsified with Freund's incomplete adjuvant and injected into thigh muscles multiple times; 28 days after this protocol, purified PGF protein was emulsified with normal saline and injected into the auricular vein. After 14 days, serum was obtained and anti-PGF antibodies were purified.

### Construction of the experimental animal model

**Figure 1:**
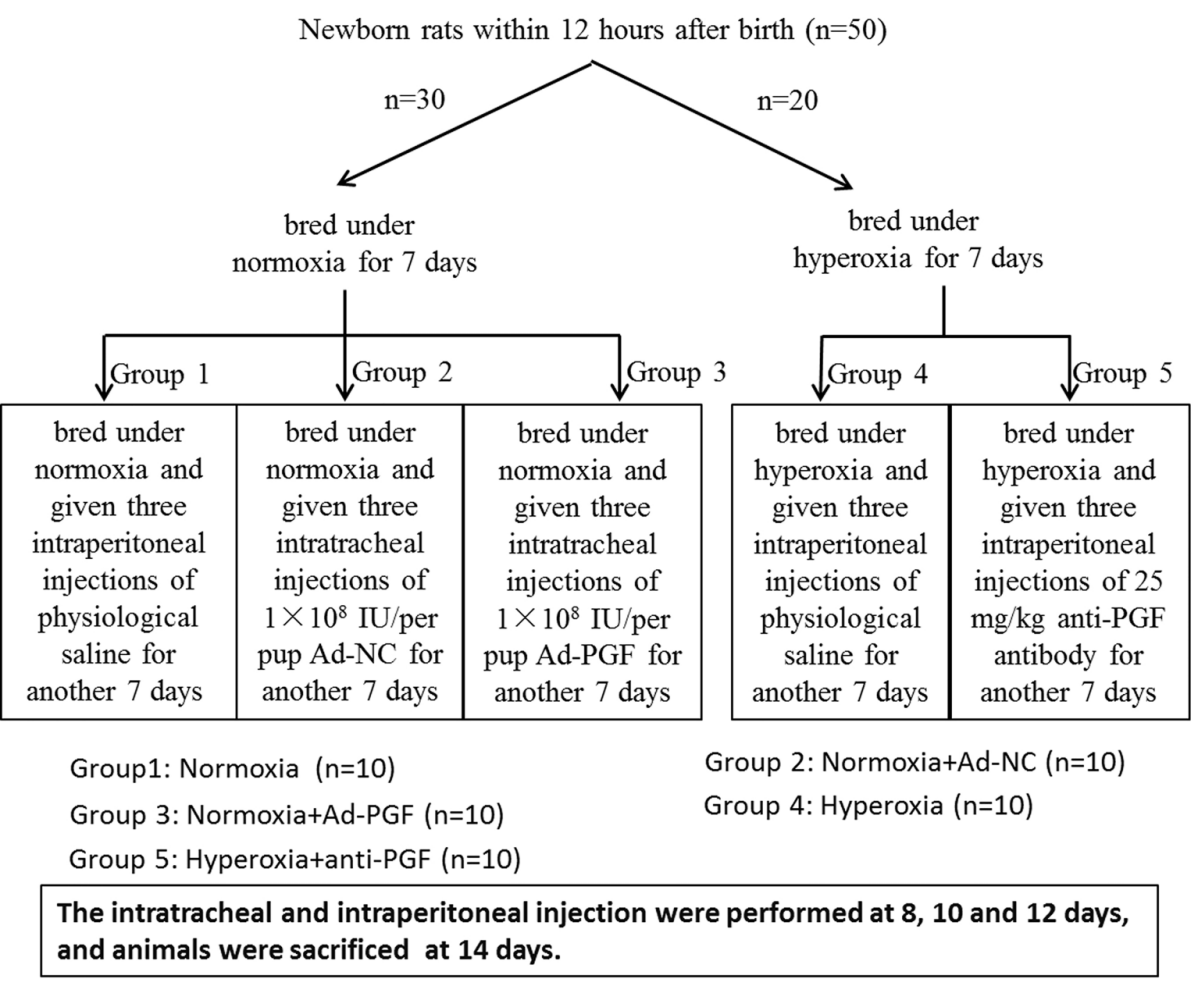
Flow chart of the method used for generating the experimental animal model. Ad-NC: adenovirus-negative control; Ad-PGF: adenovirus-placental growth factor.

Within 12 h after birth, newborn rats (the sex of the newborn rats was not ascertained) were randomly divided into Hyperoxic (n=20) and Normoxia (n=30) groups, and were exposed to hyperoxia (85% O_2_) and normoxia (21% O_2_), respectively, in a Plexiglas case with continuous O_2_ monitoring. After 7 days, the pups subjected to normoxia were divided into three groups, Normoxia group (n=10), Normoxia+Ad-NC (n=10), and Normoxia+Ad-PGF (n=10), while the pups subjected to hyperoxia were divided into two groups, Hyperoxia group (n=10) and Hyperoxia+anti-PGF group (n=10), which were then treated according to the methods shown in Figure 1. The mother rats were switched every 12 h between hyperoxic and normoxic chambers to prevent lung damage, and were given food and water *ad libitum* for 7 days.

### Collection of bronchoalveolar lavage (BAL) fluid or lung tissue

After treatment, the animals were euthanized by an intraperitoneal injection of 3% sodium pentobarbital (120 mg/kg of animal body weight) (20). Death was verified by the lack of spontaneous breathing and absent blink reflexes. Immediately after euthanization, the heart-lung block was dissected and BAL was collected in accordance with a published protocol (21,22). BAL fluid was centrifuged at 1740 *g* for 10 min at 4°C. The supernatant was collected for ELISA and cell pellets were resuspended in 20 μL saline solution for smear preparation. To collect lung samples for real-time PCR and western blot, the middle and inferior lobes of the right lung were snap-frozen in liquid nitrogen. For hematoxylin and eosin (HE) staining and immunohistochemistry (IHC), the superior lobe of the right lung was fixed in 4% formaldehyde solution.

### HE staining and IHC

Lung tissues were fixed in 4% formaldehyde solution for 3 days. After dehydration, transparency, and paraffin infiltration, paraffin-embedded lung tissues were prepared and sliced into 4-µm-thick sections. After incubation at 65°C for 12 h, deparaffinization and rehydration were carried out using a standard protocol. For HE staining, nuclei were stained with hematoxylin solution for 5 min at room temperature. Then, the sections were rinsed under running tap water and were differentiated with 0.3% acid alcohol. The sections were then rinsed under running tap water, Scott's tap water substitute, and tap water. Next, the sections were stained with eosin for 2 min at room temperature and rinsed under running tap water. Finally, the sections were air-dried and mounted on a cover-slip.

To detect CD34 expression by immunohistochemistry, the sections were incubated with 3% H_2_O_2_ at 37°C for 10 min. After washing with PBS three times, sections were incubated with 0.01 M citric acid (pH 6.0) and heated in a microwave oven for 10 min for antigen retrieval. Next, the sections were blocked with goat serum for 30 min and incubated with anti-CD34 primary antibody (1:100 dilution) (Abcam, USA) at 4°C overnight. The next day, sections were washed three times with PBS and incubated with biotinylated secondary antibody for 30 min at 37°C, followed by incubation with streptavidin-horseradish peroxidase for 5 min. After washing with PBS three times, diaminobenzidine (DAB) was added for visualization and sections were counterstained with hematoxylin. Finally, the sections were air-dried and mounted on a cover-slip. The expression of CD34 and morphological changes in these lung tissue sections were observed under a light microscope (Olympus, Japan).

The integrated absorbance of CD34 expression in images was quantified using the Image-Pro Plus 6.0 software (Media Cybernetics Inc., USA). The integrated absorbance of the Normoxia group was arbitrarily assigned a value of 1. Alveolar structures were quantified using a motorized microscope stage by employing the mean linear intercepts (MLIs) method (23,24). In brief, ten fields of every section were selected randomly and each alveolar wall was counted first in the horizontal direction and then in the vertical direction. To obtain the MLI values, an average of two numbers per field was used in the equation: Lm = (0.57/average intercepts) × 1000 (μm).

### Transmission electron microscopy

Lung tissues (<1 mm^3^) were fixed with 2.5% glutaraldehyde phosphate buffer overnight. The next day, the tissues were washed twice with 0.1 M PBS for 15 min and fixed with 1% osmic acid for 1 h. Next, they were washed twice with 0.1 M PBS (15 min each), followed by staining with uranium acetate staining solution (Sinopharm, China) for 30 min. They were then gradually dehydrated twice in 50% ethanol (Sinopharm) for 15 min, followed by 15 min in 70% ethanol, 15 min in 90% ethanol, 15 min in 100% ethanol, and 15 min in 100% acetone (Sinopharm). Then, lung tissues were infiltrated with anhydrous acetone (Sinopharm) and epoxy resin monomer (SPI Supplies Division Structure Probe, Inc., USA) at a ratio of 1:1 and incubated on an oscillating shaker for 2 h. Next, the tissues were embedded with 100% embedding agent for 2 h on an oscillating shaker. After gradual polymerization at 37°C for 24 h, 45°C for 24 h, and 60°C for 48 h, ultrathin sections (120 nm) were prepared using a microtome. Each section was mounted on a copper grid. Samples were contrasted in 4% aqueous uranyl acetate (Sinopharm) (20 min) and then in Reynolds lead citrate (Sinopharm) (5 min). Lamellar bodies and blood barriers were observed under a TECNAI-10 transmission electron microscope (Philips, Netherlands).

### Western blot analysis

Total protein was isolated from lung tissue homogenates. The lysate was centrifuged at 11,000 *g* for 1 min at 4°C. Supernatants were collected and the protein content was quantitated using a BCA Protein Assay Kit (Beyotime Institute of Biotechnology, China). Equal amounts of total protein were separated using 12% SDS polyacrylamide gels, and then transferred on to PVDF membranes (Millipore, USA). After blocking with 5% milk in TBS containing 0.05% Tween-20 (TBST) for 1 h at 37°C, membranes were incubated with rabbit anti-actin monoclonal antibody (1:1000 dilution, ab179467, Abcam), rabbit anti-VEGF Receptor 1 monoclonal antibody (1:2000 dilution, ab32152, Abcam), or rabbit anti-PGF polyclonal antibody (1:1000 dilution, TA332424, OriGene Technologies, USA) at 37°C for 1 h. After washing with TBST three times, all membranes were incubated with goat anti-rabbit IgG H&L (HRP) preadsorbed secondary antibody (1:5000 dilution) (Abcam) at 37°C for 40 min. After washing with TBST three times, protein expression was visualized using Immobilon Western Chemilum HRP Substrate (Millipore). Actin served as an internal loading control. Relative protein expression was calculated using a method described previously (25).

### Enzyme-linked immunosorbent assay (ELISA)

The rat interleukin-6 (IL-6) ELISA kit (HEA079Ra) and rat tumor necrosis factor (TNF)-α ELISA kit (SEA133Ra) (Wuhan USCN Business Co., Ltd., USA) were used to measure IL-6 and tumor necrosis factor (TNF)-α levels in the BAL fluid.

### Statistical analysis

All results are reported as means±SD, and were analyzed using SPSS version 19.0 software (IBM, USA). Intergroup comparisons were conducted using one-way analysis of variance (one-way ANOVA), followed by *post hoc* tests of the least significant difference for multiple pairwise comparisons. P values <0.05 were considered statistically significant.

## Results

### Effect of anti-PGF antibody treatment on PGF and Flt1 expression in the lung tissues of hyperoxia-treated newborn rats

**Figure 2:**
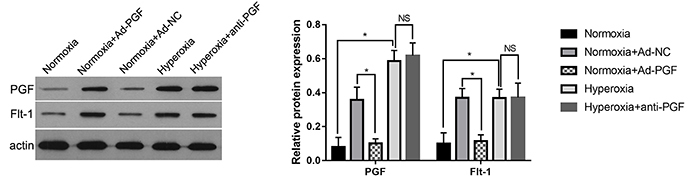
Placental growth factor (PGF) and Flt1 expression in the lung tissues of newborn rats of each group (n=10) detected by western blot analysis. Actin served as the internal loading control. Representative western blots (left) and statistical results of relative protein expression (right) are shown. Data are reported as means±SD. *P<0.05 (ANOVA). Ad-NC: adenovirus-negative control; Ad-PGF: adenovirus-placental growth factor; NS: not significant.

The lung tissues of newborn rats exposed to the respective experimental conditions were collected and western blot analysis was performed to observe the expression of PGF and Flt1. It was observed that there was a significant increase in the expression of PGF and Flt1 in the lung tissues of the Normoxia+Ad-PGF group compared to that in the Normoxia+Ad-NC group (Figure 2). In addition, the expression of PGF and Flt1 in the lung tissues of the Hyperoxia group was higher than that in the Normoxia group. Moreover, the PGF and Flt1 levels in the lung tissues of the Hyperoxia+anti-PGF group were the same as those in the Hyperoxia group, indicating that the anti-PGF antibody did not have a significant effect on the expression of PGF and Flt1.

### Effect of anti-PGF antibody on the development of pulmonary alveoli in hyperoxia-treated newborn rats

**Figure 3:**
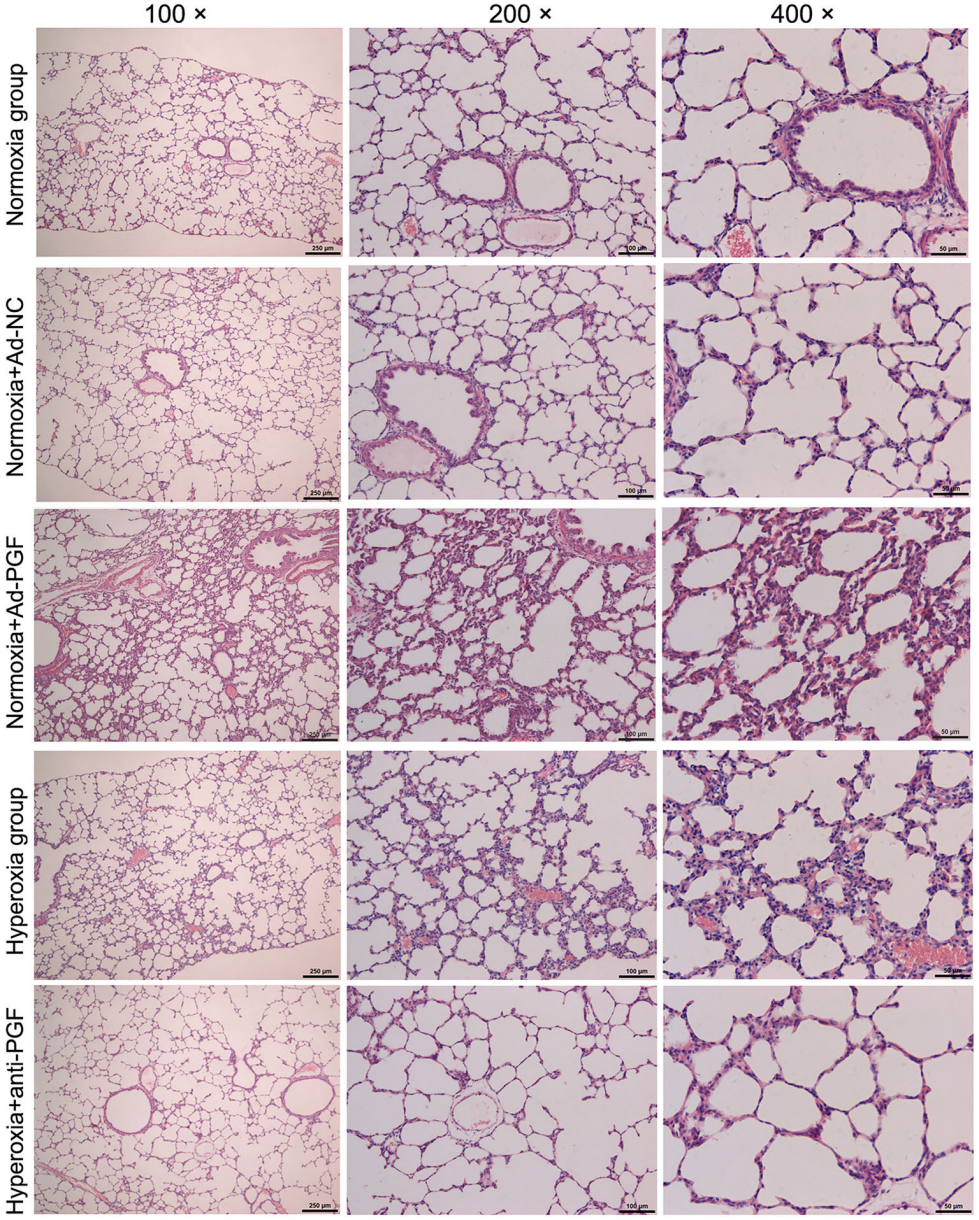
Representative histology of lung tissues of newborn rats of each indicated group (n=10) detected by hematoxylin and eosin staining. Magnifications: 100, 200, and 400×; scale bars: 250, 100, and 50 μm. PGF: placental growth factor; Ad-NC: adenovirus-negative control; Ad-PGF: adenovirus-placental growth factor.

Lung tissues of newborn rats exposed to different experimental treatments were collected and evaluated using HE staining. It was observed that tissues from rats in the Normoxia and Normoxia+Ad-NC groups showed normal histology (Figure 3). Damaged alveoli and thickened alveoli septa were observed in the lung tissues of newborn rats in the Normoxia+Ad-PGF and Hyperoxia groups. However, this hyperoxia-induced damage was alleviated by intraperitoneal injection of anti-PGF antibody.

**Figure 4:**
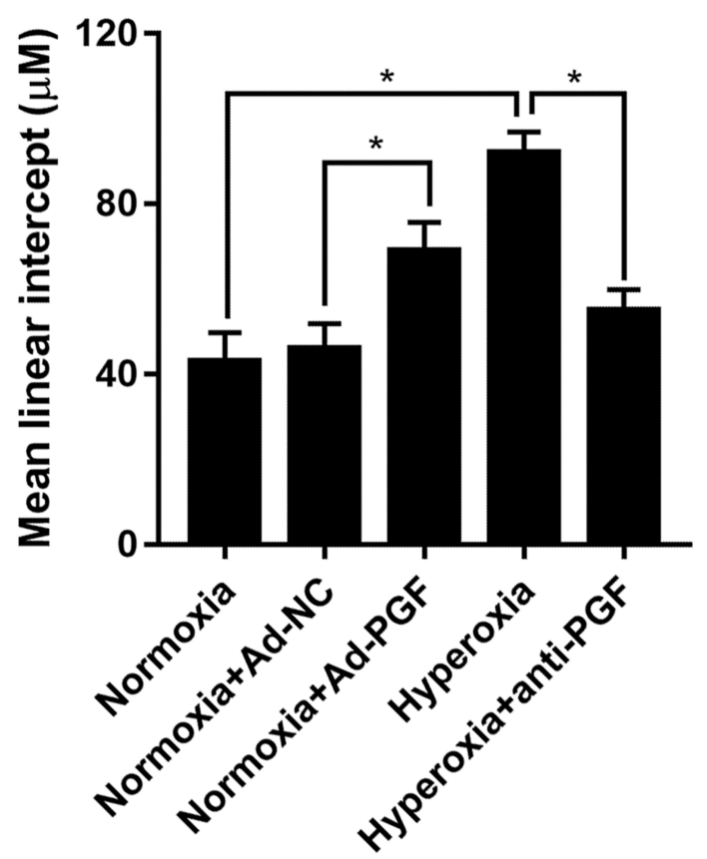
Mean linear intercept values (MLIs) of lung tissues from each group of newborn rats (n=10). Data are reported as means±SD. *P<0.05 (ANOVA). PGF: placental growth factor; Ad-NC: adenovirus-negative control; Ad-PGF: adenovirus-placental growth factor.

Furthermore, MLIs were measured for a more accurate assessment of the effect on pulmonary alveoli development. The lungs of newborn rats in the Normoxia+Ad-PGF group exhibited significantly higher MLIs than those in the Normoxia+Ad-NC group (Figure 4). In addition, the lungs of rats from the Hyperoxia group also exhibited significantly higher MLIs than those in the Normoxia group. Further, the anti-PGF antibody treatment significantly reduced the hyperoxia-induced increase in the MLI value.

### Effect of anti-PGF antibody on the ultrastructure of alveolar epithelial cells in hyperoxia-treated newborn rats

**Figure 5:**
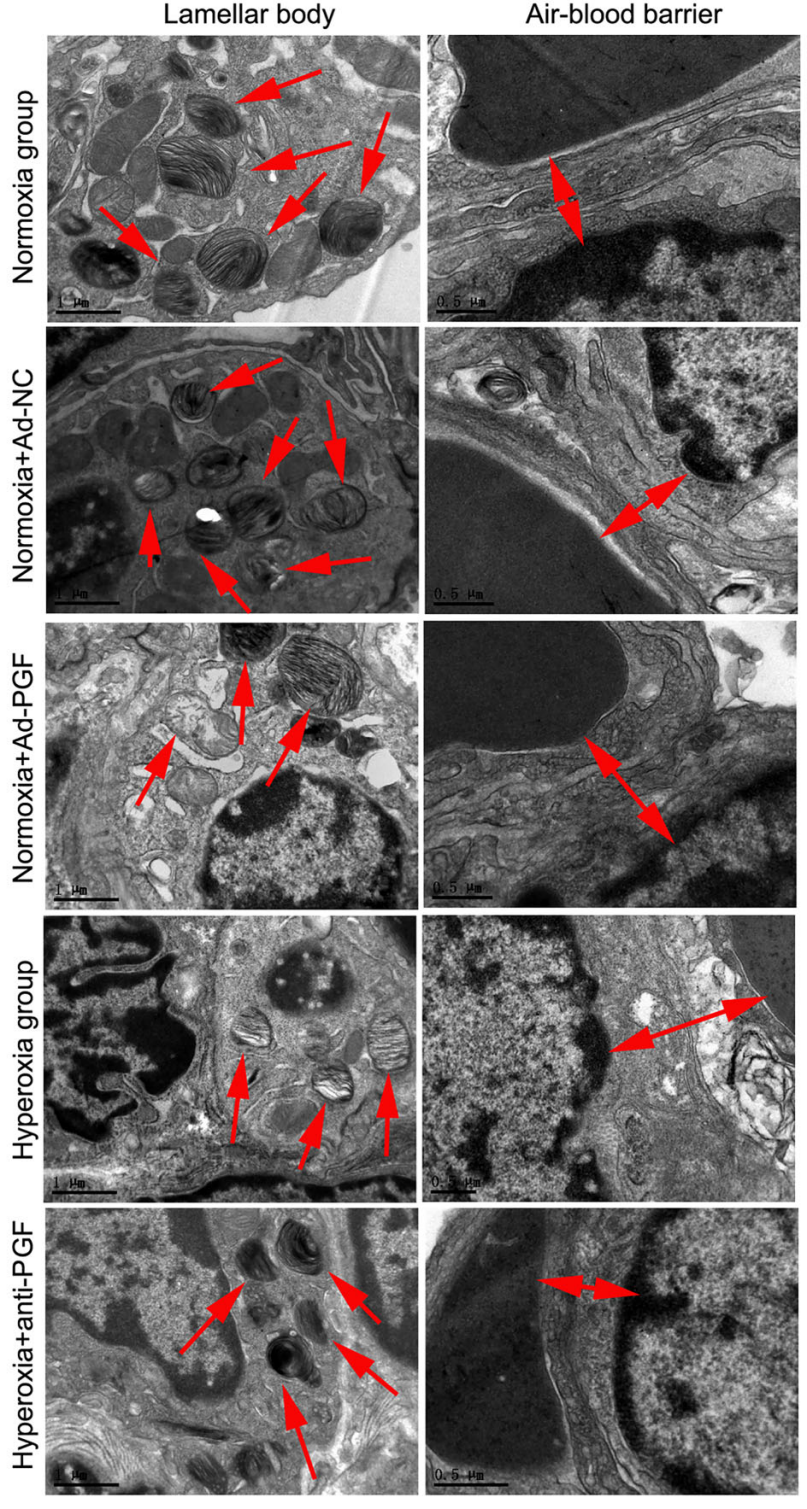
The ultrastructure of alveolar epithelial cells (arrows) of newborn rats of each indicated group (n=10). Scale bars in left images: 1 μm; scale bars in right images: 0.5 μm. PGF: placental growth factor; Ad-NC: adenovirus-negative control; Ad-PGF: adenovirus-placental growth factor.

Lung tissues of newborn rats from different experimental groups were collected and the ultrastructure of alveolar epithelial cells was evaluated using transmission electron microscopy (Philips, The Netherlands) (Figure 5). Lamellar bodies of alveolar epithelial cells in the Normoxia and Normoxia+Ad-NC groups were uniform in size and number. However, lamellar bodies with vacuole-like abnormalities appeared in alveolar epithelial cells of the Normoxia+Ad-PGF and Hyperoxia groups. In addition, the air-blood barriers in the alveolar epithelial cells were thin and tight in the Normoxia and Normoxia+Ad-NC groups, while those in the Normoxia+Ad-PGF and Hyperoxia groups were wider and leaky. These hyperoxia-induced changes to the ultrastructure of lamellar bodies and air-blood barriers of alveolar epithelial cells were alleviated by the intraperitoneal injection of the anti-PGF antibody.

### Effect of anti-PGF antibody on IL-6 and TNF-&mac_alpha; levels in BAL fluid of hyperoxia-treated newborn rats

**Figure 6:**
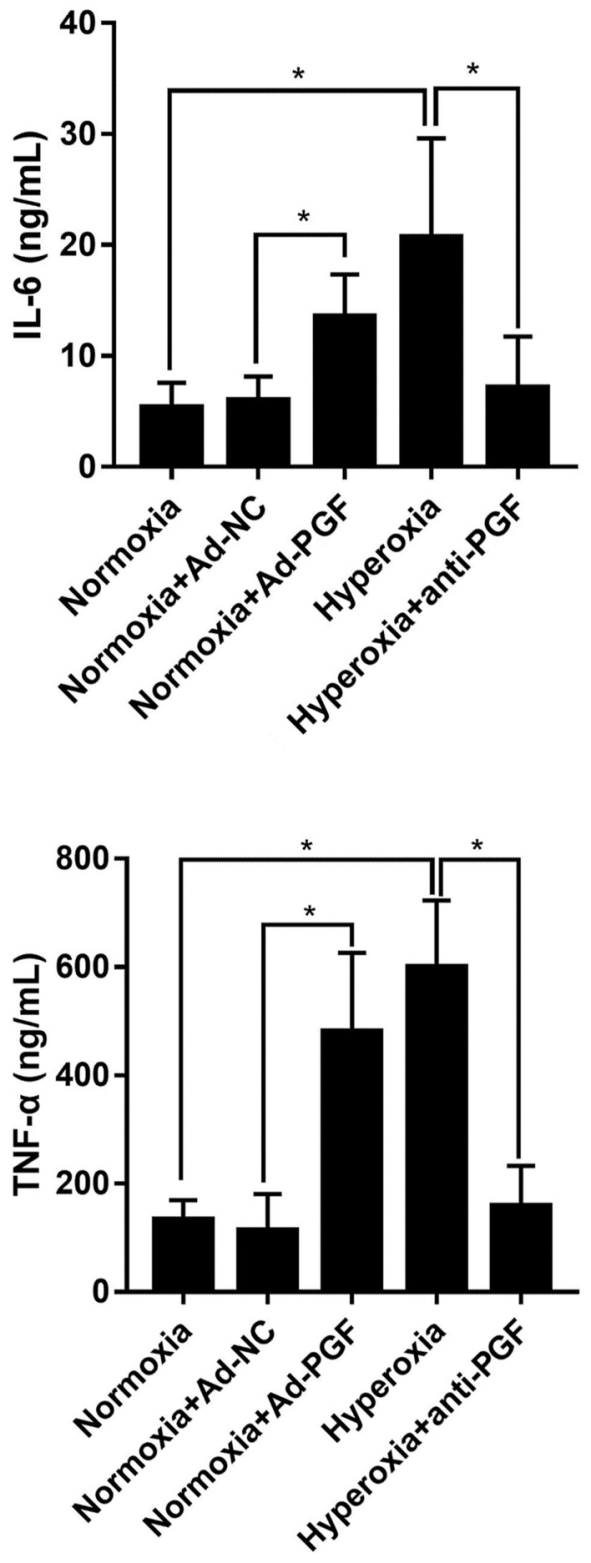
Interleukin (IL)-6 and tumor necrosis factor (TNF)-α levels in bronchoalveolar lavage fluid from newborn rats of each indicated group (n=10) detected by ELISA. Data are reported as means±SD. *P<0.05 (ANOVA). PGF: placental growth factor; Ad-NC: adenovirus-negative control; Ad-PGF: adenovirus-placental growth factor.

BAL fluid was collected from newborn rats in each experimental group and an ELISA was performed to detect IL-6 and TNF-α levels. The levels of IL-6 and TNF-α were found to be significantly increased in the BAL fluid of the Normoxia+Ad-PGF group compared to those in the Normoxia+Ad-NC group (Figure 6). In addition, IL-6 and TNF-α levels in the BAL fluid of rats from the Hyperoxia group were higher than those in rats from the Normoxia group. However, the IL-6 and TNF-α levels in BAL fluid of the Hyperoxia+anti-PGF group were significantly decreased compared to those in the Hyperoxia group.

### Effect of treatment with anti-PGF antibody on CD34 expression in the lung tissues of hyperoxia-treated newborn rats

**Figure 7:**
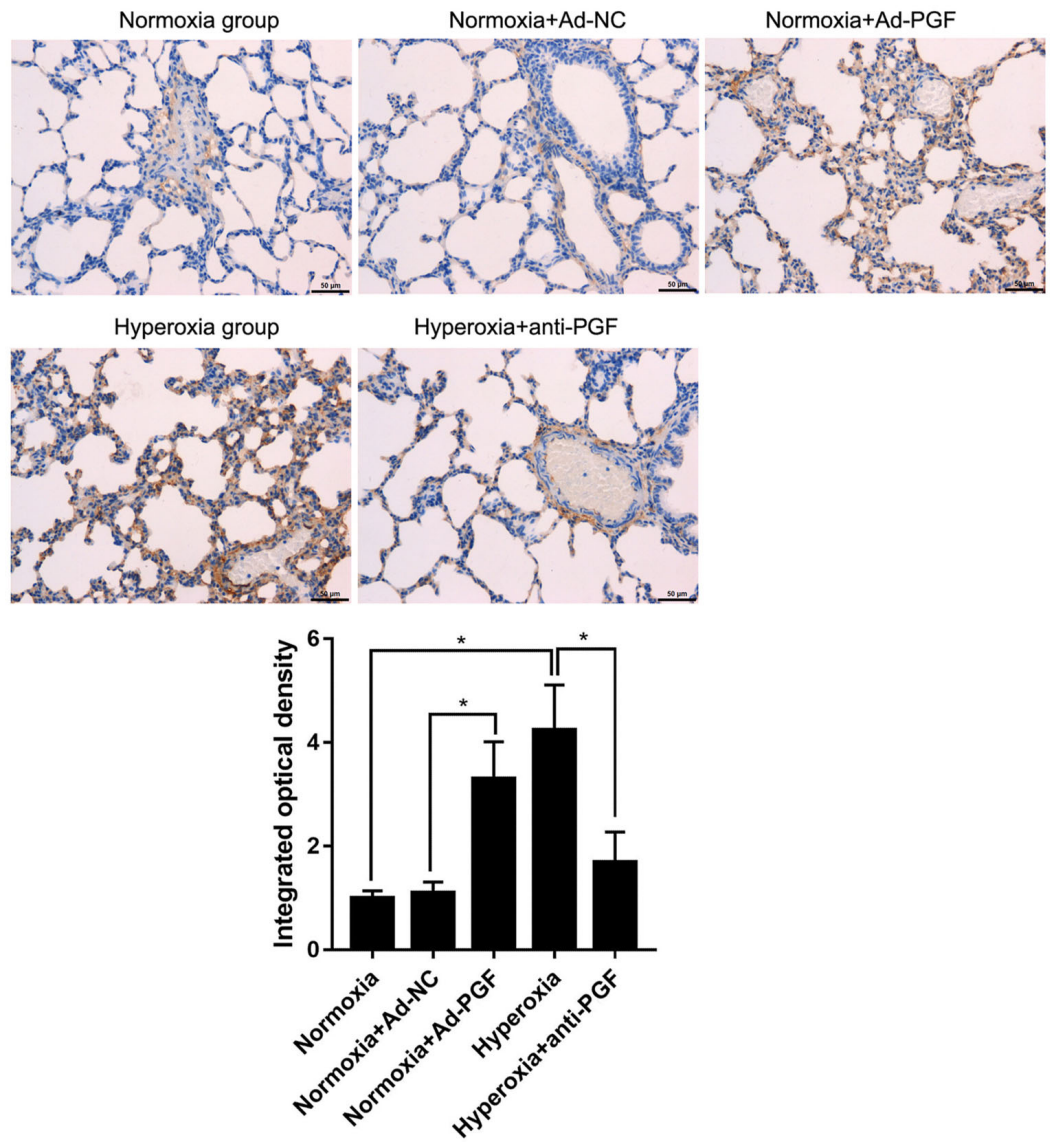
CD34 expression detected by immunohistochemistry in lung tissues from newborn rats of each indicated group (n=10) quantified using the Image-Pro Plus 6.0 software. Magnification: 200×; scale bar: 50 μm. The integrated optical density of the Normoxia group was arbitrarily set as 1. Data are reported as means±SD. *P<0.05 (ANOVA). PGF: placental growth factor; Ad-NC: adenovirus-negative control; Ad-PGF: adenovirus-placental growth factor.

Lung tissues of newborn rats were collected and IHC was carried out to evaluate CD34 expression. CD34 levels were found to be significantly increased in the lung tissues of the Normoxia+Ad-PGF group compared to that in the Normoxia+Ad-NC group (Figure 7). In addition, CD34 expression in the lung tissues of rats from the Hyperoxia group was higher than that in rats from the Normoxia group. In contrast, CD34 expression in the lung tissues of the Hyperoxia+anti-PGF group was significantly lower than that in the Hyperoxia group.

## Discussion

Studies have shown that PGF expression could be increased by hyperoxia (18), and PGF knockdown could improve hyperoxia-induced acute lung injury in neonatal rats (19). However, there is no report regarding the direct investigation of the role of PGF overexpression on lung development in newborn rats under normoxic conditions. This limits the ability to fully understand the role of PGF in hyperoxia-induced lung injury and related diseases. In the present study, we found that, in a manner similar to hyperoxia, PGF overexpression could increase MLI, disrupt the ultrastructure of alveolar epithelial cells, promote aberrant pulmonary vascular growth, and induce inflammatory responses in neonatal rats. These results indicated that PGF overexpression impaired lung development in newborn rats. Thus, PGF may serve as a target for the development of therapeutic drugs to treat hyperoxia-induced lung injury and related diseases such as BPD.

Our results indicated that injecting anti-PGF antibody does not reduce the hyperoxia-induced abnormally high expression of PGF and its receptor Flt1, but significantly recovers lung tissue injury, as well as reduces the expression of TNF-α and Il-6 in BAL, and CD34 in lung tissues. The results suggested that blocking the action of PGF by an anti-PGF antibody ameliorated the damage resulting from hyperoxia exposure. Our study thus advocates that anti-PGF antibodies can be potentially used for the clinical management of bronchopulmonary dysplasia in premature infants, and can have translational significance.

It is known that PGF levels are low or undetectable in healthy tissue but are increased in pathological conditions (26,27). We have found that hyperoxia induced PGF expression, which is consistent with the findings of previous reports (28). However, the levels of PGF and its receptor Flt1 in the Hyperoxia+anti-PGF group were not decreased compared to those in the Hyperoxia group. These results indicated that the anti-PGF antibody does not affect the expression of PGF and its receptor Flt1. It is known that antibodies bind antigens based on antigen recognition by the variable region of the antibody (29). Hence, we predicted that the anti-PGF antibody and PGF protein could form an antibody-antigen complex, thereby decreasing the binding of PGF to its receptor Flt1, ultimately blocking the downstream signaling pathways. In addition, the anti-PGF antibody may induce global changes in the lung proteome, which might trigger the PGF-independent protective effect against hyperoxia-induced lung injury. However, detailed investigations are needed to understand the relationship between antibody-antigen complex formation and the mechanisms underlying the protective effect of anti-PGF antibody on hyperoxia-induced lung injury.

Hyperoxic lung injury is primarily characterized by damaged and decreased number of alveoli, local atelectasis, and thickened alveoli septa (30). Treatment with anti-PGF antibody significantly reduced the hyperoxia-induced increase in MLI. In addition, intraperitoneal injection of anti-PGF antibody alleviated hyperoxia-induced disruption in the ultrastructure of alveolar epithelial cells, such as lamellar bodies with vacuole-like abnormalities and wider air-blood barriers. Taken together, these results revealed that intraperitoneal injection of the anti-PGF antibody alleviated hyperoxia-induced lung damage. Our conclusion is in agreement with a previous study by Zhang et al. (19), who reported that knocking down PGF by injecting lentiviral particles containing PGF-specific shRNA via the temporal vein mitigates hyperoxia-induced acute lung injury in neonatal rats. Long-term exposure to hyperoxia is one of the critical factors in the development of BPD (13). Therefore, anti-PGF antibodies may have a therapeutic potential in the context of BPD. Our results point towards a new therapeutic approach for the treatment of BPD.

The pro-inflammatory effects of PGF have been observed in various cells and organs (31–33). Therefore, the level of inflammatory cytokines can also be measured to verify the function of the anti-PGF antibody. Hyperoxia-mediated overproduction of pro-inflammatory cytokines such as IL-6 and TNF-α in the BAL fluids has been verified in a previous study (19), as well as in the present study. In addition, it has been reported that PGF knockdown suppresses the expression of pro-inflammatory cytokines IL-6 and TNF-α (19,31); the findings of our present study are consistent with those of these reports. We found that the levels of IL-6 and TNF-α in BAL fluid of the Hyperoxia+anti-PGF group were significantly decreased compared to those in the Hyperoxia group. These results indicated that the anti-PGF antibody played an anti-inflammatory role, which was similar to the effect of PGF knockdown (19,31). Overproduction of TNF-α and IL-6 by macrophages has been linked to several inflammatory diseases, such as BPD (34,35). Thus, strategies to downregulate the expression of these pro-inflammatory mediators during inflammation are considered to be potential therapeutic tools to alleviate the progression of inflammatory diseases, such as BPD (35). These results further demonstrated the therapeutic potential of anti-PGF antibodies in the context of BPD.

Moreover, it has been reported that the loss of PGF impairs angiogenesis during ischemia, inflammation, wound healing, and cancer, indicating that PGF functions as an angiogenic factor (36). These reports reveal that PGF is a key regulatory factor involved in regulating angiogenesis in pathological conditions (37). Additionally, BPD is characterized by aberrant pulmonary vascular growth and remodeling (4–6). Therefore, we evaluated the expression of CD34, a marker of angiogenesis, to further demonstrate the potential therapeutic role of the anti-PGF antibody in BPD. Our results indicated that the CD34 levels in lung tissues of the Hyperoxia+anti-PGF group were significantly decreased compared to those in the Hyperoxia group. These findings indicated that the anti-PGF antibody attenuated hyperoxia-induced aberrant pulmonary vascular growth, thereby demonstrating the potential therapeutic role of anti-PGF antibodies in BPD. In addition, it is reported that PGF induces angiogenesis by stimulating the migration and proliferation of the microvascular endothelium (38). Therefore, we predicted that the anti-PGF antibody inhibits aberrant pulmonary vascular growth by preventing the effect of PGF on migration and proliferation of endothelial cells from bovine coronary postcapillary venules and from human umbilical veins, which may indicate the possible cellular mechanism underlying the effect of anti-PGF antibody on angiogenesis.

In a future study, we will examine the effect of sex factors and administration routes on the role of anti-PGF antibody for the treatment of abnormal development of pulmonary vasculature and alveoli in hyperoxia-exposed neonatal rats. This aspect was a limitation of this study and it should be therefore investigated in future research. In addition, the lung tissues were embedded in paraffin, which affects the optimal structural preservation. Moreover, we will focus on the regulatory mechanisms by measuring mRNA, ncRNA, and protein levels to further evaluate the potential therapeutic role of anti-PGF antibodies in BPD.

In conclusion, PGF overexpression and hyperoxia could increase MLI, disturb the ultrastructure of alveolar epithelial cells, increase aberrant pulmonary vascular growth, and promote inflammatory responses in neonatal rats. Intraperitoneal injections of an anti-PGF antibody were shown to alleviate hyperoxia-induced lung damage, overproduction of TNF-α and IL-6 in BAL fluids, and aberrant pulmonary vascular growth. These results indicated that PGF overexpression impaired lung development in newborn rats. Blocking the action of PGF with an anti-PGF antibody ameliorated hyperoxia-mediated impairments in lung development in neonatal rats. These findings, for the first time, suggested that anti-PGF antibodies exhibited therapeutic potential for BPD, and pointed towards a new treatment method for BPD.
